# Supplementary material for: Bacterial Amino Acid Auxotrophies Enable Energetically Costlier Proteomes
Source: Int J Mol Sci. 2025 Mar 4;26(5):2285. doi: 10.3390/ijms26052285 (PMC11900164; doi:10.3390/ijms26052285)
Supplement: Supplementary file 1 [file ijms-26-02285-s001.zip › Figure S1 Starke auxotrophies.pdf]

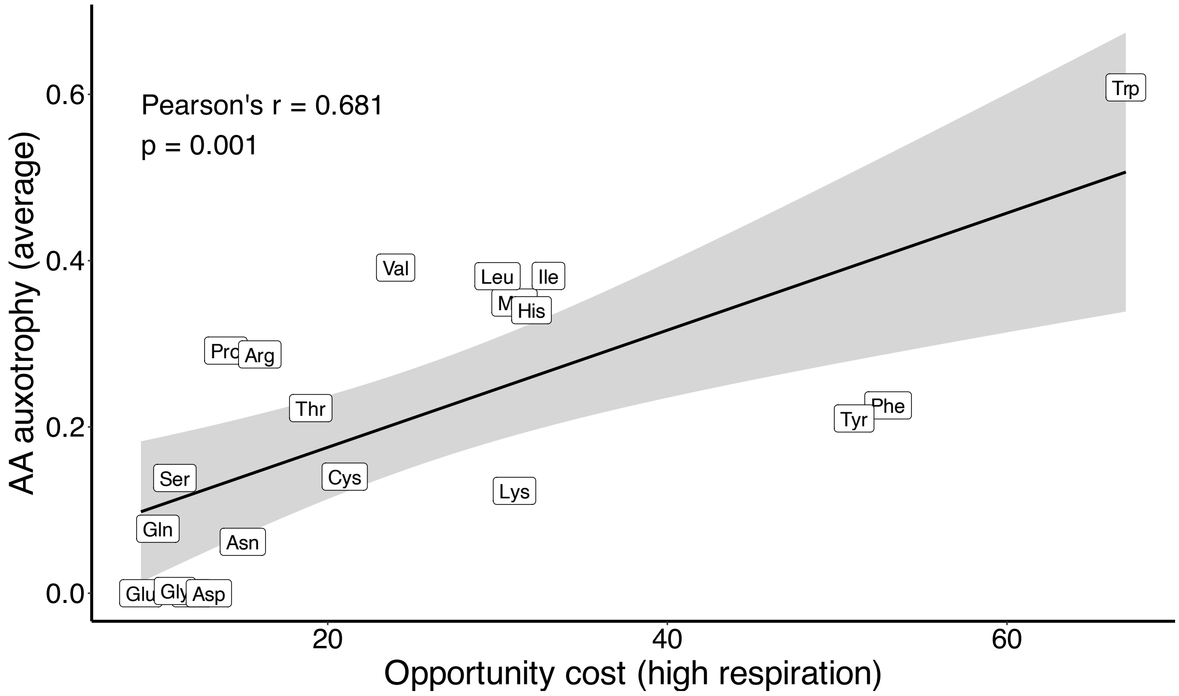

Figure S1. Correlation between AA biosynthesis cost and the average AA auxotrophy based on data from Starke et al. 2023 [14]**.** We calculated the average of AA auxotrophy binary values provided for 3,687 bacterial species in the Starke et al. 2023 [14]. This value was then correlated with the opportunity cost of each AA, previously calculated for high respiration (see Materials and Methods). The Pearson correlation coefficient and *p*-value are displayed on the graph. Amino acids are marked using three-letter codes: Alanine (Ala), Arginine (Arg), Asparagine (Asn), Aspartic acid (Asp), Cysteine (Cys), Glutamic acid (Glu), Glutamine (Gln), Glycine (Gly), Histidine (His), Isoleucine (Ile), Leucine (Leu), Lysine (Lys), Methionine (Met), Phenylalanine (Phe), Proline (Pro), Serine (Ser), Threonine (Thr), Tryptophan (Trp), Tyrosine (Tyr), and Valine (Val).
