# Supplementary material for: Bacterial Amino Acid Auxotrophies Enable Energetically Costlier Proteomes
Source: Int J Mol Sci. 2025 Mar 4;26(5):2285. doi: 10.3390/ijms26052285 (PMC11900164; doi:10.3390/ijms26052285)
Supplement: Supplementary file 1 [file File S1 Fully resolved tree with AA auxotrophy heatmap.pdf › File S1 Fully resolved tree with AA auxotrophy heatmap.pdf]

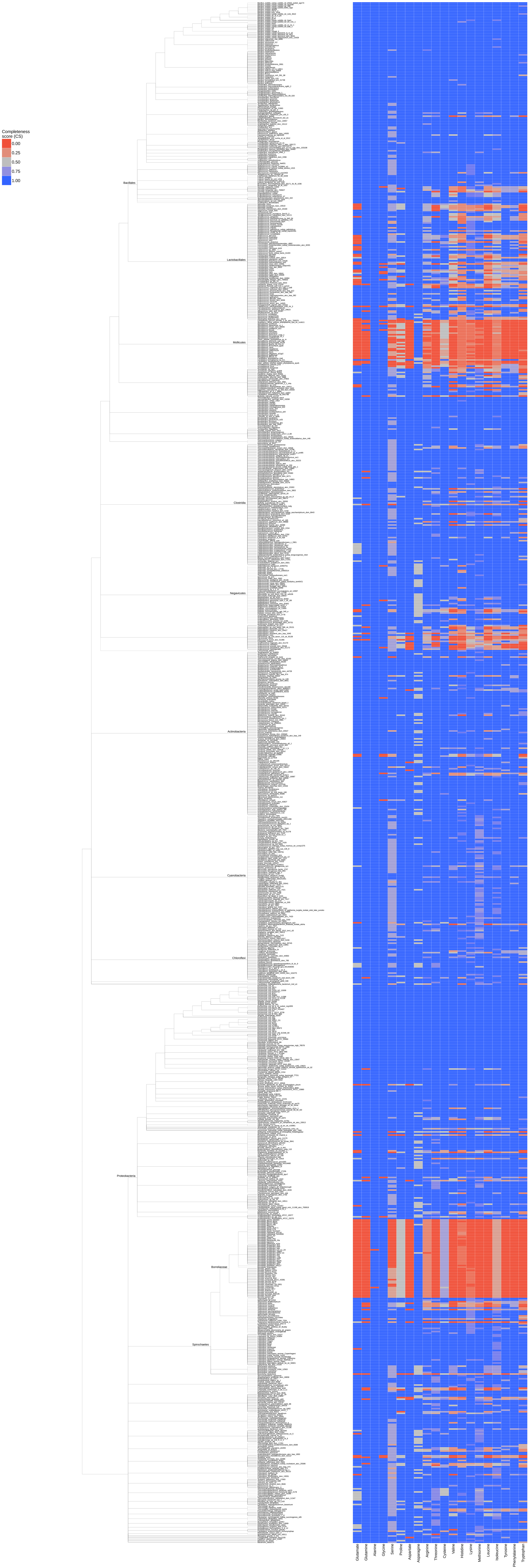
Completeness score (CS)
0.00
0.25
0.50
0.75
1.00
Bacillales
Lactobacillales
Mollicutes
Clostridia
Negativicutes
Actinobacteria
Cyanobacteria
Chloroflexi
Proteobacteria
Borreliaceae
Spirochaetes
Glutamate
Glutamine
Alanine
Glycine
Serine
Proline
Aspartate
Asparagine
Arginine
Threonine
Cysteine
Valine
Histidine
Lysine
Methionine
Leucine
Isoleucine
Tyrosine
Phenylalanine
Tryptophan
